# Anti-Erythropoietin Antibody Associated Pure Red Cell Aplasia Resolved after Liver Transplantation

**DOI:** 10.1155/2015/286276

**Published:** 2015-07-09

**Authors:** Annie K. Hung, Jennifer Guy, Caroline M. Behler, Eugene E. Lee

**Affiliations:** ^1^Department of Internal Medicine, California Pacific Medical Center, San Francisco, CA 94115, USA; ^2^Department of Hepatology, California Pacific Medical Center, San Francisco, CA 94115, USA; ^3^Department of Hematology Oncology, California Pacific Medical Center, San Francisco, CA 94115, USA

## Abstract

Patients undergoing antiviral therapy for chronic hepatitis C often develop anemia secondary to ribavirin and interferon. Recombinant erythropoietin has been used to improve anemia associated with antiviral therapy and to minimize dose reductions, which are associated with decreased rates of sustained virologic response. A rare potential side effect of recombinant erythropoietin is anti-erythropoietin antibody associated pure red cell aplasia. In chronic kidney disease patients with this entity, there have been good outcomes associated with renal transplant and subsequent immunosuppression. In this case, a chronic liver disease patient developed anti-erythropoietin associated pure red cell aplasia and recovered after liver transplantation and immunosuppression. It is unclear whether it is the transplanted organ, the subsequent immunosuppression, or the combination that contributed to the response. In conclusion, anti-erythropoietin associated pure red cell aplasia is a serious complication of erythropoietin therapy, but this entity should not be considered a contraindication for solid organ transplantation.

## 1. Introduction

Pure red cell aplasia (PRCA) is a rare normochromic, normocytic anemia with near to complete absence of erythroblasts in the bone marrow. Current standard diagnostic criteria of this condition include a decrease in red cell count of about 1% per day, reticulocyte count below 1%, and the absence of major changes in white cell count, platelet count, and differential leukocyte count [1]. Elevated serum transferrin saturation and ferritin can also be seen and reflect decreased utilization of iron stores given the absence of erythropoiesis. Confirmation of the diagnosis requires bone marrow examination which should demonstrate normal cellularity with myeloid cells and megakaryocytes, but less than 1% erythroblasts with occasionally up to 5% proerythroblasts or basophilic erythroblasts [1]. Congenital PRCA is rare and includes Diamond Blackfan anemia. There are many causes of acquired PRCA which include parvovirus B19, lymphoproliferative disorders, systemic autoimmune disease, production of erythropoietin-neutralizing antibodies, and drugs such as chloramphenicol [2].

Since their introduction in 1989, erythropoiesis stimulating agents (ESA) have been used widely to improve anemia in those with chronic renal failure [1]. After prolonged use, patients can develop anti-erythropoietin antibodies which also have neutralizing capacity for endogenous erythropoietin, leading to PRCA. This is a rare entity with the estimated overall exposure-adjusted incidence of 0.02–0.03 per 10,000 patient-years [3]. It is associated with a minimum exposure to ESA for 3 weeks, usually occurring after 6 to 8 months [3]. Most cases have been associated with subcutaneous administration [1]. Incidence of antibody-mediated PRCA from intravenous administration is exceedingly rare [3].

Combinations of interferon and ribavirin have long been the standard of treatment for chronic hepatitis C. A common side effect of interferon is bone marrow suppression which can affect granulocytes, erythrocytes, and megakaryocytes [4]. Ribavirin has been known to cause a dose dependent hemolysis, by triggering intracellular ATP depletion leading to cell membranes that are more prone to oxidative damage [5]. When undergoing treatment with combination therapy of ribavirin and interferon, dose reductions are often necessary because of anemia [4]. In severe cases, antiviral therapy has to be discontinued [4]. Studies have also demonstrated an association between dose reductions and decreased sustained virologic response (SVR) [6]. Large randomized trials have shown that administration of epoetin alfa in hepatitis C patients receiving interferon and ribavirin therapy helped with anemia, resulting in fewer dose reductions, and improved quality of life [7, 8].

## 2. Case Report

A 66-year-old African American man underwent treatment for chronic hepatitis C while awaiting liver transplantation due to hepatocellular carcinoma. He was started on triple therapy with telaprevir 750 mg every 8 hours, ribavirin 1000 mg daily, and pegylated interferon 180 mcg subcutaneously weekly. Two weeks into treatment, he developed anemia with drop in hemoglobin to 10 g/dL, from his baseline of 13 g/dL. The anemia was attributed to ribavirin and thus the dose was reduced to 400 mg twice daily. He was also started on epoetin alfa 40,000 units subcutaneously per week.

He underwent a thorough work-up for his anemia including parvovirus serologies, upper endoscopy and colonoscopy, hemoglobin electrophoresis, iron studies, folate, vitamin B12, HIV, ANA, and RPR which were all negative. Because of persistent anemia, his ribavirin dose was again decreased to 200 mg daily. His epoetin alfa dose was increased to 60,000 units per week without any substantial response. After 5 months of recombinant erythropoietin therapy, he became transfusion dependent, requiring 2 to 3 units of packed red blood cells every 3 to 4 weeks, receiving over 30 units over a 7-month period. Because of his severe anemia, ribavirin and peg interferon were both discontinued.

**Figure 1:**
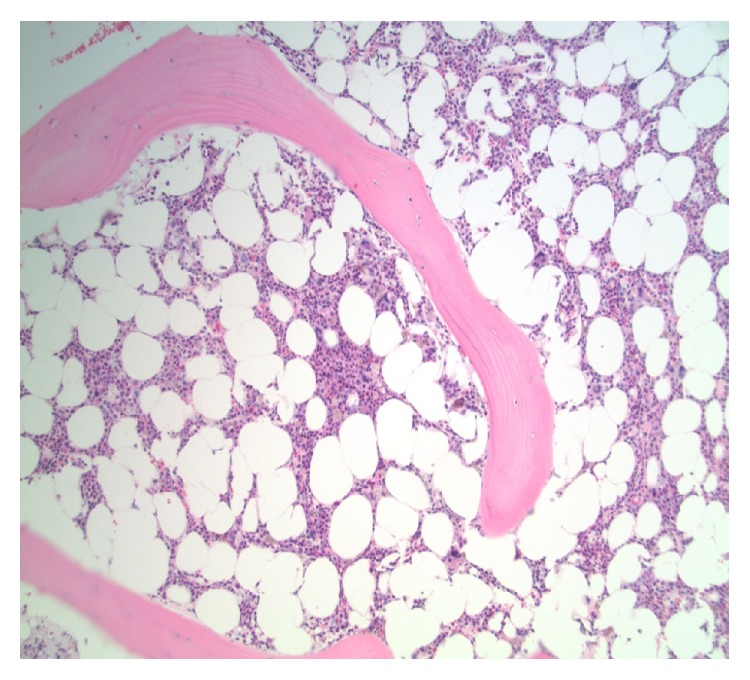
Bone marrow showing hypocellularity and trilineage hematopoiesis, without increased blasts. No increased fibrosis or dysplastic changes are seen.

**Figure 2:**
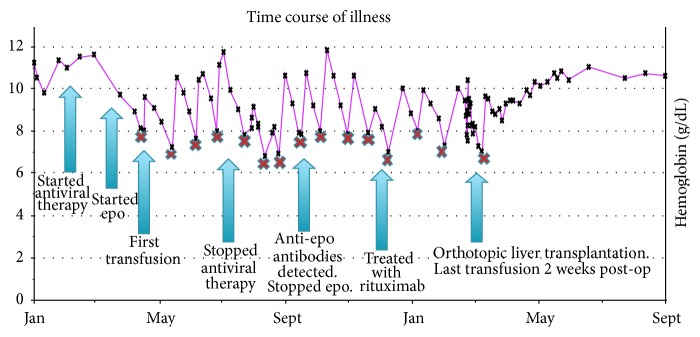
A graph of fluctuating hemoglobin levels throughout time course of illness. The patient experienced a drop in hemoglobin shortly after starting antiviral therapy. Despite dose reduction and discontinuation of antivirals, he was transfusion dependent. Packed red cell transfusions are marked by the red X. After orthotopic liver transplantation, hemoglobin levels increased without any further transfusion requirements. He has continued to have sustaining hemoglobin levels >10 g/dL.

A bone marrow biopsy showed absence of reticulocytes, along with 15% cellularity, with trilineage hematopoiesis (Figure 1). There were no dysplastic findings or increase in fibrosis, but he did have increased marrow stores. Because he had been treated with erythropoietin without much increase in his blood count and remained transfusion dependent, an assay to detect anti-erythropoietin antibodies was sent and found to be positive. The assay showed neutralizing capacity of 21.4 mcg per mL, predominantly IgG1 with minor component of IgG2 and IgG4. Epoetin alfa was immediately discontinued. The patient was felt to have pure red cell aplasia due to anti-erythropoietin antibodies. The hypocellularity seen on bone marrow examination was thought to be related to interferon and ribavirin therapy. He was started on rituximab 375 mg per meters squared intravenously weekly and received 4 treatments. However, after cessation of epoetin alfa and antiviral therapy, as well as completion of rituximab, he continued to be transfusion dependent. The included graph depicts his fluctuating hemoglobin levels throughout the course of his illness (Figure 2).

The patient was listed for liver transplantation for hepatocellular carcinoma but had become inactive because of his anemia. He had uncomplicated Child-Pugh class A cirrhosis with no history of portal hypertension. Despite his anemia, he had completed an adequate course of antiviral therapy and had sustained virologic response at 6 months, presumed to be cured of hepatitis C. Based on lesions measuring 3.6 cm and 1.7 cm, he was also within the Milan criteria. The decision was made to undergo orthotopic liver transplantation. It was also felt that chronic immunosuppression after transplantation would help his anemia. He underwent transplantation without complications. Immunosuppressive therapy was started with tacrolimus, mycophenolate, and prednisone. After liver transplantation and chronic immunosuppression, his anemia improved with stable hemoglobin and reticulocyte levels. Ten weeks after transplantation, his hemoglobin was greater than 10.0 g/dL. He has not required any further transfusions. Repeat titer did not detect anti-erythropoietin antibodies.

## 3. Discussion

Most cases of antibody associated PRCA have occurred in patients with chronic kidney disease. The majority of these cases were associated with a preparation of epoetin alfa marketed outside the United States (Eprex/Erypo) [3]. Following concerns for transmission of Creutzfeldt-Jacob disease, human serum albumin was removed from Eprex and replaced by polysorbate 80 and glycine [3]. It has been proposed that the polysorbate 80 formulation was less stable, causing aggregation of epoetin alfa molecules especially at increased temperatures, increasing immunogenicity [3]. Another hypothesis proposes that leachates from uncoated rubber syringe stoppers in prefilled syringes could have also increased immunogenicity, leading to antibody formation [3]. Because of the concern for increasing immunogenicity, the subsequent formulations of Eprex replaced uncoated rubber stoppers with fluororesin-coated stoppers and the polysorbate was removed [3].

Although antibody associated PRCA is a serious complication of recombinant erythropoietin therapy, screening for the presence of anti-erythropoietin antibodies is not justified without clinical suspicion [9]. The 2012 Kidney Disease: Improving Global Outcomes guidelines suggest testing for anti-erythropoietin antibodies after exposure of at least 8 weeks to an ESA, decline in hemoglobin level of >0.5 to 1.0 g/dL per week, or transfusion requirement of at least 1 to 2 units per week, normal white blood cell and platelet count, and absolute reticulocyte count <10,000/microL [9]. The most accurate test to detect anti-erythropoietin antibodies is the radioimmunoprecipitation assay, but it is time consuming and difficult to automate [10]. Enzyme-linked immunosorbent assays are not as accurate but are widely available [11].

The therapeutic recommendations for antibody associated PRCA are cessation of ESA therapy, correction of anemia with blood transfusions as needed, and prompt initiation of immunosuppressive therapy [3]. Typical starting doses for immunosuppressive therapies are 0.5–1.0 mg/kg/day for corticosteroids and 200 mg/day for cyclosporine [3]. In a retrospective review of 47 patients with erythropoietin-induced PRCA, all patients developed this entity after receiving epoetin alfa (Eprex, Ortho Biotech) [12]. Median delay between initiation of erythropoietin treatment and start of pure red cell aplasia was 11 months [13]. Recovery rates were between 56% and 88% in patients treated with corticosteroids, cyclophosphamide, or cyclosporine [13]. However, there were 6 patients who underwent kidney transplant who all recovered from transfusion dependence within 1 month [13]. In other reports from Europe and Canada in 1999 and 2000, several patients with antibody associated PRCA also underwent renal transplantation after failing immunosuppressive regimens and had rapid clinical recovery [11]. In several countries, policies were changed to make patients with antibody associated PRCA a higher priority on waiting lists for kidney transplantation [14]. The data is limited, but kidney transplantation appears to be an effective treatment for this condition [14]. However, it is unclear whether the transplanted organ or the subsequent immunosuppression is the therapeutic agent.

There have been several reports of PRCA associated with chronic hepatitis C and its treatment. Most cases are antibody-mediated and associated with epoetin alfa. In one case, a 65-year-old woman developed antibodies after epoetin alfa treatment for anemia induced by ribavirin and interferon for hepatitis C therapy and was treated successfully with rituximab [13]. In a similar case, a 50-year-old man developed antibody associated PRCA after receiving epoetin alfa for anemia secondary to ribavirin and interferon and was treated successfully with danazol [15]. There are also several cases in the literature of patients with chronic hepatitis C who developed PRCA but did not receive recombinant erythropoietin [14]. Anti-erythropoietin antibodies were not tested in these patients, but the etiology of the PRCA was thought to be the hepatitis C virus itself versus drug induced by either ribavirin or interferon [14].

There is limited literature on PRCA and liver transplant. As far as we know, there are no other cases of this entity resolving after liver transplantation. There have been documented cases of PRCA secondary to parvovirus B19 infection after liver transplant and immunosuppression which responded well to immunoglobulin [16]. Interestingly, there is a case reported of a patient who underwent liver transplantation, started antiviral therapy for hepatitis C with ribavirin and interferon, received recombinant erythropoietin for anemia, but developed anti-erythropoietin antibody-mediated PRCA while on immunosuppression [17]. As mentioned above, immunosuppression should minimize antibody production, but in this patient increased immunogenicity caused by interferon therapy may have led to antibody associated PRCA [17].

The question of rechallenging patients who have recovered from antierythropoietin mediated PRCA is controversial. There have been multiple case reports of patients who have been successfully treated with immunosuppressive therapy and then rechallenged with another epoetin [18]. Rechallenging should be considered if anti-erythropoietin antibodies are around or below the limit of detection and with careful monitoring of reticulocyte and hemoglobin levels [19]. If a patient is rechallenged, administration should be intravenous [19].

Peginesatide is a synthetic dimeric peptidic ESA which activates the human erythropoietin receptor but its primary amino acid sequence is unrelated to human erythropoietin. Thus, it does not induce an immune response against endogenous erythropoietin, decreases the risk of antibody-mediated PRCA, and has been proposed as an alternative to immunosuppression [20]. Based on two randomized controlled trials (EMERALD 1 and EMERALD 2), peginesatide was approved by the US FDA for treatment of anemia in hemodialysis patients [20]. Shortly after FDA approval, peginesatide was recalled [20]. As of now, the application of peginesatide to treat antibody-mediated PRCA is not clear.

## 4. Conclusion

Antibody associated PRCA is a rare but serious complication of recombination erythropoietin therapy. It should be considered when a patient continues to be anemic despite treatment with recombinant erythropoietin products. A wide range of immunosuppressive therapies as well as kidney transplantation have been associated with clinical recovery of this entity. There are still many unanswered questions regarding the root causes of antibody associated PRCA, as well as the long term outcomes of patients with this entity who have undergone solid organ transplantation with liver and kidney.

## Abbreviations

ESA: Erythropoiesis stimulating agents
PRCA: Pure red cell aplasia
SVR: Sustained virologic response
ATP: Adenosine triphosphate.
